# Study of the Single-Screw Extrusion Process Using Polylactide

**DOI:** 10.3390/polym15193878

**Published:** 2023-09-25

**Authors:** Mariusz Fabijański

**Affiliations:** Plastics Processing Department, Faculty of Mechanical and Industrial Engineering, Warsaw University of Technology, 85 Narbutta Street, 02-524 Warsaw, Poland; mariusz.fabijanski@pw.edu.pl

**Keywords:** plastic extrusion, polylactide, PLA, process analysis

## Abstract

This study presents the extrusion process while using a single-screw extruder and polylactide (PLA). This material belongs to the so-called biodegradable plastics, and is characterized by a higher density compared to typical polymeric materials used to manufacture products in this technology. Various polyethylenes and polypropylenes and their derivatives are commonly used. An evaluation of the extrusion process was carried out for various extruder operating parameters. The rotational speed of the screw and the process temperature were changed. For each rotational speed of the screw, the following readings were made: changes in temperature, active power, current intensity, pressure, and mass of extruded plastics each time.

## 1. Introduction

Plastic extrusion is one of the main technologies used to produce plastic products. As a result of this process, so-called continuous products in the form of profiles, plates, or pipes are obtained. This technology is also used to manufacture composite products such as reinforced hoses for transporting liquids or gases and electric wires. This process also produces packaging [1,2,3,4,5,6].

This process is quite well known and described, but research is still being carried out on modeling this process, both for the plastics themselves and those filled with organic fillers (wood flour, cellulose fibers) and mineral fillers (calcium carbonate, glass fibers) [6,7,8,9,10], among other substances. To sum up, this process consists of plasticizing the material in the plasticizing system of the extruder, and then shaping it by passing it through a properly shaped head, which is an open tool [11,12].

Plastics in the form of granules, powders, flakes, or chips are used as the starting material. Due to the technological process itself, the plasticizing mechanism and its rheological properties, various screw designs as well as boring heads are used, in which appropriate changes in geometry are introduced [13,14,15,16,17,18,19,20].

Polylactide (PLA) was used in the study of the extrusion process. It is classified as biodegradable plastic and is a promising polymer for this type of application. It can be produced annually with a capacity of more than 140,000 tons. Low greenhouse gas emissions and low energy consumption are also notable factors [21,22,23]. Substances of mineral origin, such as calcium carbonate and others, can be successfully added to PLA as fillers [24,25,26,27,28].

Products made from this material can be produced using the technology of injection, extrusion, and 3D printing [22,23,24,25]. Compared to typical polymers intended for these production technologies, it is characterized by a higher density, ranging from 1.20 g/cm^3^ to about 1.35 g/cm^3^ depending on the type. Typical plastics (PP, PE, PS, ABS, and others) have a density of 0.9 g/cm^3^ to 1.05 g/cm^3^. This parameter is not without influence on the processing and production of products, both in the technology of injection, extrusion, or at least the increasingly popular 3D printing. In this case, this material is one of the most popular raw materials [29,30,31,32,33,34,35,36,37,38,39,40,41,42,43,44].

This paper attempts to assess the PLA extrusion process depending on the process conditions, such as screw rotational speed and temperature. Studying the impact of these parameters on the extrusion process is important, both from the point of view of optimization of the production process, and the future development of computer models for designing and simulating this process. The study of the impact of screw rotational speed and temperature on the PLA extrusion process aims to understand how these variables affect the quality and properties of the final product. For example, temperature has a key influence on a plastic’s viscosity, flowability, and other rheological properties that affect the process.

Literature analysis in this area provides important information on the optimal ranges of temperatures and rotational speeds for traditional polymers (PE, PP), which lead to the best results in the extrusion process. The results obtained in this work can be used to develop a computer model that will predict the behavior of PLA under various conditions of this technological process. Such a model can be extremely valuable when designing new production processes, optimizing existing ones and anticipating potential problems.

The undoubted novelty of this development is the comprehensive approach that takes into account both the extruder system and the head tool.

## 2. Materials and Methods

In this work, polylactide (PLA) by NatureWorks (USA) under the name Ingeo Biopolymer Ingeo 4043D was used for the research, and it is intended for processing extrusion technology. Table 1 gives basic data on this material.

**Figure 1:**
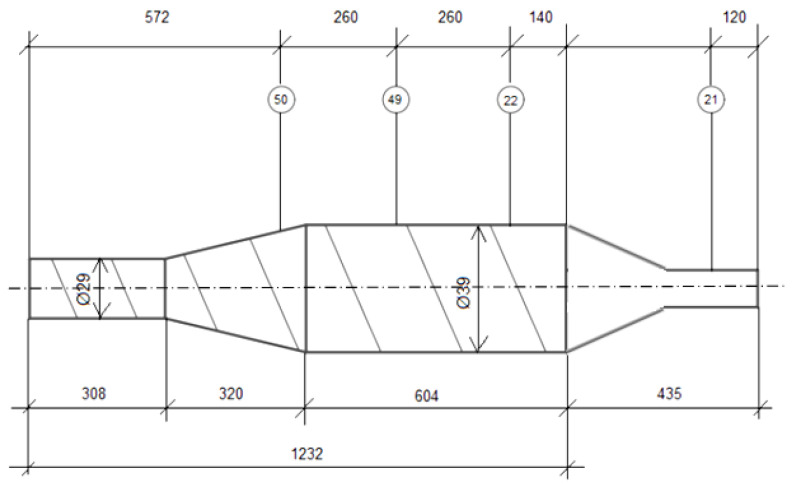
The general geometry of the screw and die and the location of the pressure sensors.

**Figure 2:**
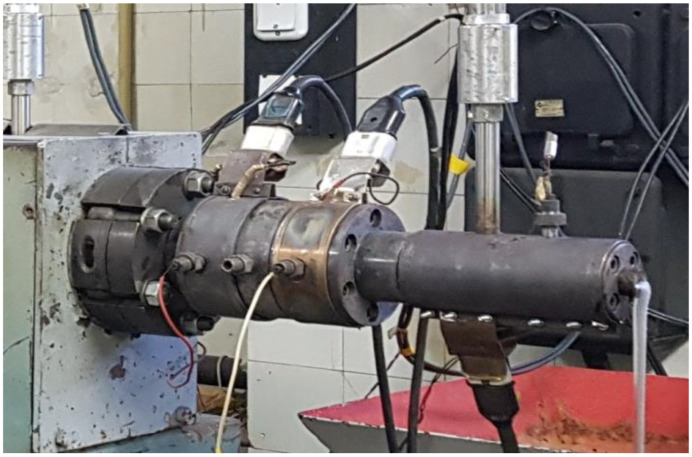
Die photo.

Experimental studies of the single-screw extrusion process were carried out on a single-screw extruder T-45 manufactured by Metalchem (Poland), equipped with a screw with a diameter of D = 45 mm and a ratio of L/D = 27. It has four heating zones equipped with temperature sensors. As a tool, an extrusion die with three heating zones was used to form a flat profile with a width of 20 mm and a thickness of 2 mm. In the second zone it was armed with a pressure sensor. Figure 1 shows a diagram illustrating the geometry of the coping screw and the die, as well as the arrangement of the pressure sensors. Figure 2 shows a photo of the die.

In addition, the station was equipped with an electronic DARwag scale, a KC 100/200 dryer and a grinding mill. PLA was dried in a drawer dryer at 90 °C for 48 h in order to eliminate water that could affect the measurements.

The tests of the extrusion process were carried out for various operating parameters of the extruder. The rotational speed of the screw and the process temperature were changed. Process temperatures were T1 = 180 °C and T2 = 200 °C, respectively. The rotational speed of the screw also changed to n1 = 20 rpm; n2 = 40 rpm; n3 = 60 rpm; n4 = 80 rpm, respectively. During the process, PLA was extruded at each temperature, and speed and three samples were cut every 15 s to measure the weight. For each rotational speed of the screw during the process, readings were also made of changes in temperature for each of the four zones: active power (P), current (I), pressure on individual sensors (p), and the previously mentioned mass of three successive samples at an interval of time15 s.

After all measurements, calculations were made and individual parameters were compared, such as: viscosity for different temperatures, mass extruded during the process, mass yield, pressure drop, volumetric flow, and shear rate.

## 3. Results and Discussion

Table 2 presents the results of pressure changes depending on the rotational speed of the screw on three sensors located on the extruder in given zones, and the pressure on the extrusion die. These parameters refer to the process temperature T1 = 180 °C. The increase in pressure in individual zones of the plasticizing system is clearly visible. This is confirmed by the generally accepted mechanism in this type of process, where the material is transported, plasticized, and mixed in individual zones. The lower die pressure also confirms this mechanism. The task of this tool is to cause the appropriate pressure to drop so that it flows freely at the end and does not pulsate.

Table 3 shows the measurement result of the active power and the current drawn by the motor for the process temperature T1 = 180 °C. With the increase in rotational speed, a clear increase in current consumption can be seen, and thus the active power increases. This is due to the resistance of the material during the plasticization process at a lower temperature.

Table 3 also shows the mass of material samples taken during 15 s. A fourfold increase in the rotational speed of the screw results in an increase in the mass of extrudate by three and a half times. The same applies to the process efficiency related to the processing of PLA in one hour.

The next step was to repeat the experiment for the process temperature T2 = 200 °C at the same set of rotational speeds. The pressure, active power, amperage, and mass of the extruded samples were read in the same way for 15 s. Table 4 presents the values of the read pressure on individual sensors in the system plasticizing and on the die. As in the case of processing at the temperature of 180 °C (Table 2), an increase in pressure is observed with the length of the screw and a decrease at the die. At the processing temperature of 200 °C, slightly lower pressure values were also observed in the individual zones of the screw and the die.

Table 5 presents the cumulative results of the active power measurement, the amperage drawn by the motor for the process temperature T2 = 200 °C. Compared to the process carried out at the temperature T1 = 180 °C, a reduced demand for amperage, and thus active power, during the process is observed (Table 3). Generally, the resistance during processing at a higher temperature is lower, and the material flows more easily in the plasticizing system of the extruder and in the die itself. This also affects the measured pressure values.

After carrying out the necessary measurements, the necessary calculations characterizing the extrusion process were made. Among other things, viscosity and shear rate were determined for individual temperatures T1 and T2 and screw rotational speeds adopted in the experiment.

The calculations were carried out in accordance with the formulas used to describe the parameters of the extrusion process [12,13,14]. Table 6 presents the results of calculations for individual screw revolutions and the temperature T1 = 180 °C, while Table 7 presents the results of calculations for the temperature T2 = 200 °C.

**Figure 3:**
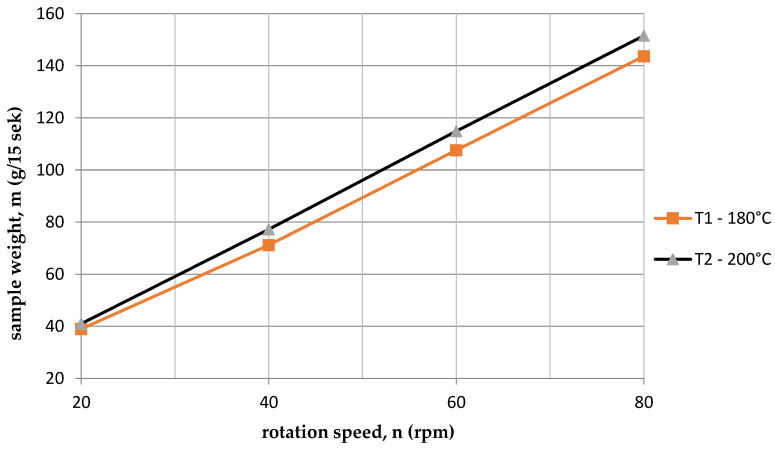
Dependence of the sample mass on the rotational speed of the screw for two temperatures.

A graphical comparison of the results is shown in the respective subsequent figures. Figure 3 (Table 6 and Table 7) shows a comparison of the dependence of the mass on the rotational speed of the screw, depending on the processing temperature. It can be said that this relationship is linear. The higher the rotational speed of the screw, the more material is transported. The amount of material here is affected by the temperature. A higher temperature causes a faster transition to a liquid state and the viscosity is lower, which improves fluidity and reduces resistance during the extrusion process.

**Figure 4:**
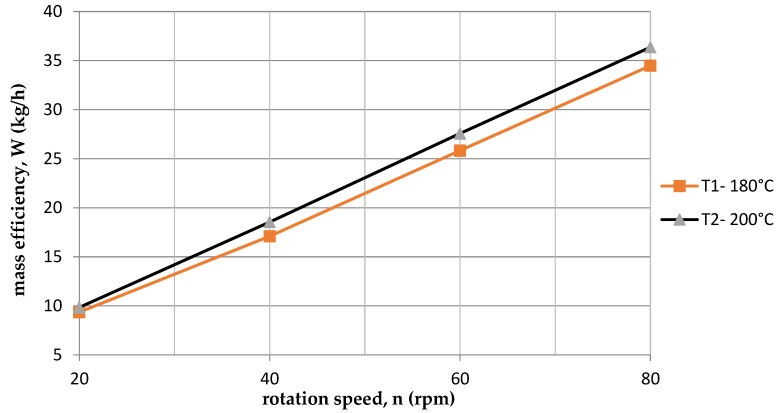
Dependence of mass efficiency on screw speed for two temperatures.

The same applies to performance (Figure 4, Table 6 and Table 7). It is higher for the temperature T2 = 200 °C. With the increase of the rotational speed of the screw, the mass of the sample and mass efficiency increase. At the second temperature T2 = 200 °C, a greater mass of samples was obtained, and thus a higher mass yield W compared to the first temperature T1 = 180 °C (Table 6 and Table 7).

**Figure 5:**
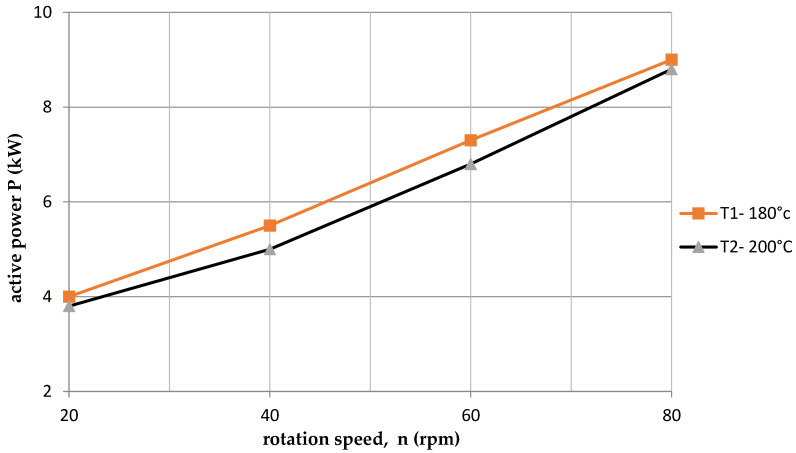
The dependence of active power on the screw speed for two temperatures.

Figure 5 shows the dependence of the power consumption P on the rotational speed of the screw n. It was noticed that the active power values are higher for the lower temperature T1 = 180 °C. In this case, the so-called “clogging” of the screw contributed to the formation of higher resistance. Higher material viscosity occurred at a lower temperature. In the case of a higher temperature T2—200 °C, the resistance was much lower, and PLA was more fluid.

**Figure 6:**
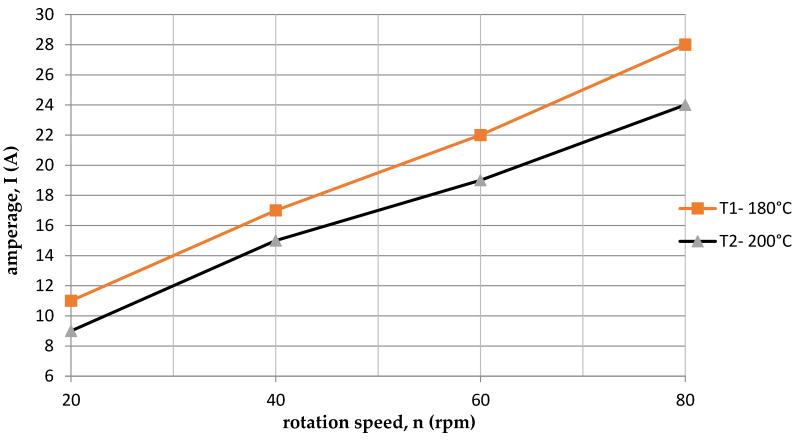
Dependence of the amperage intensity on the rotational speed of the screw for two temperatures.

**Figure 7:**
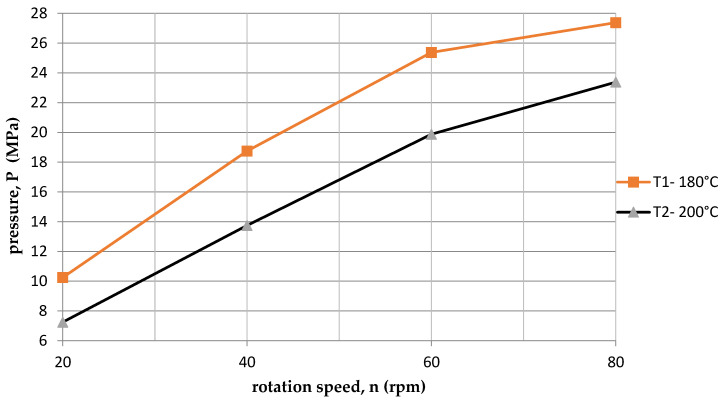
The dependence of the pressure on the rotational speed of the screw for two temperatures.

On the other hand, Figure 6 shows the dependence of the current intensity I on the rotational speed of the screw n for two temperatures (T1 = 180 °C and T2 = 200 °C). The amperage was higher for the first temperature T1 = 180 °C. This was because at the lower temperature T1 = 180 °C, there were higher resistances than at the process temperature T2 = 200 °C. It is also directly related to the change in pressure. Figure 7 shows a comparison of the dependence of the pressure p and the rotational speed of the screw for two temperatures. The pressure p was higher for the first temperature T1 = 180 °C, and the pressure p increased with the increase in screw revolutions n.

**Figure 8:**
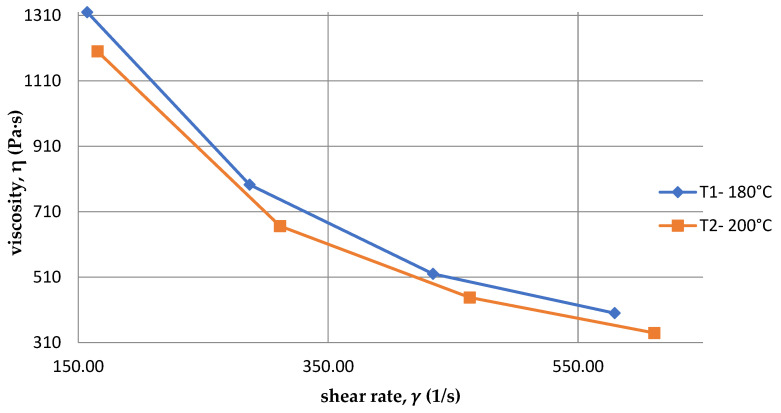
PLA viscosity curves for two different temperatures.

Figure 8 show the dependence of the viscosity η on the shear rate 𝛾 of PLA at two temperatures. It is easy to see that at a higher temperature T2 = 200 °C, the viscosity of the material is lower compared to the temperature T1 = 180 °C. This is because as the temperature increases, the kinetic energy of the molecules and the distances between them increase in accordance with the definition of viscosity. It is also a measure of internal friction, so as the distance between particles increases, its value decreases. This property applies to all polymers.

**Figure 9:**
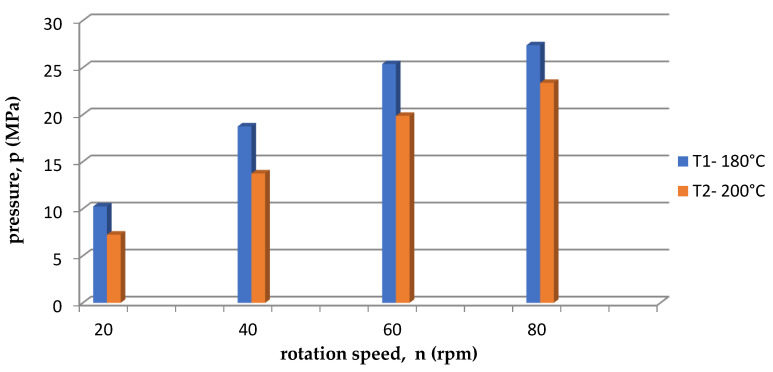
Average pressure for each temperature.

Analyzing the data contained in Table 6 and Table 7, the values of the mean pressure p for the first measurement T1 = 180 °C were slightly higher compared to the second measurement for the temperature T2 = 200 °C. This is graphically presented in Figure 9. In addition, the viscosity value was higher for the temperature of T1 = 180 °C, so it can be concluded that the viscosity of PLA increased simultaneously with the increase in pressure.

**Figure 10:**
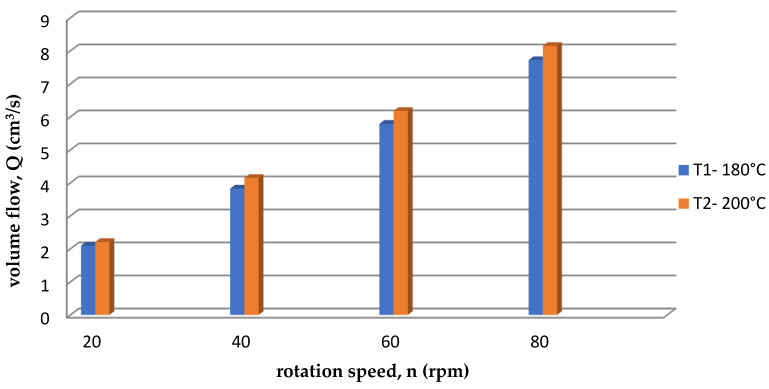
Volume flow for each temperature.

During the first measurement for T1 = 180 °C, it was observed that the volumetric flow was lower compared to the second measurement of T2 = 200 °C. This is illustrated by the graph in Figure 10. This is because polylactide was extruded at a lower temperature to the so-called “clogging” of the screw, and it hindered the flow. At a higher temperature (in the considered case T2 = 200 °C) there was no “clogging of the screw”, so the flow was smoother and the volumetric flow rate was higher.

Analyzing the graphs in Figure 9 and Figure 10, it can be concluded that the viscosity of polylactide increased with increasing pressure and temperature, and decreased with increasing volumetric flow rate. These are characteristics of almost all polymers.

The obtained test results coincide with the theoretical information on processing in the extrusion method. It can be concluded that PLA behaves like other polymers during extrusion.

**Figure 11:**
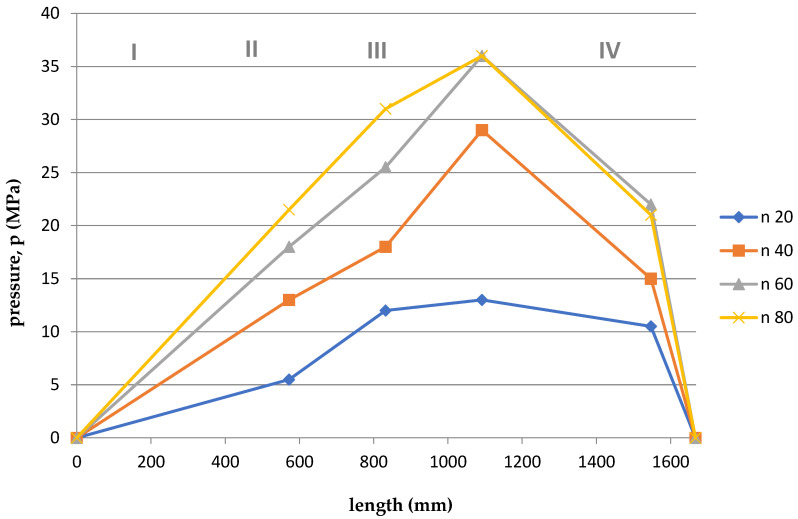
The course of pressure changes during extrusion for the first measurement. Where: I—supply zone, II—compression zone, III—dosing zone, IV—die.

**Figure 12:**
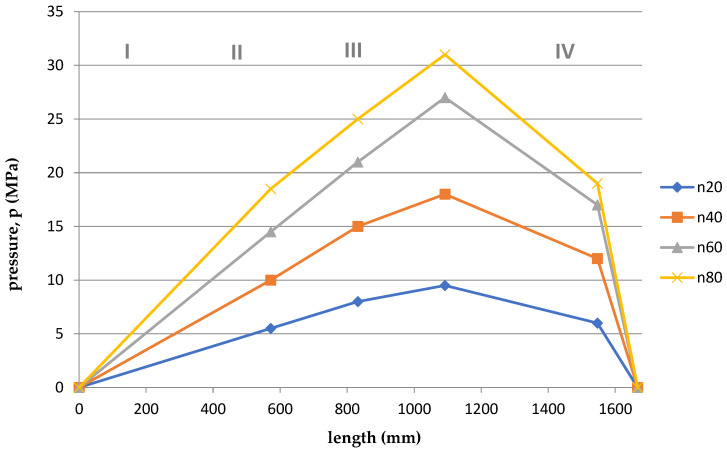
The course of pressure changes during extrusion for the second measurement. Where: I—supply zone, II—compression zone, III—dosing zone, IV—die.

**Figure 13:**
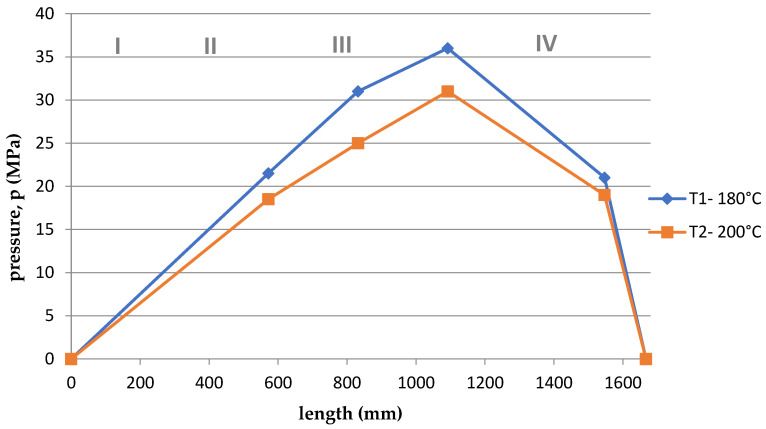
The course of pressure changes during extrusion for two temperatures at 80 rpm. Where: I—supply zone, II—compression zone, III—dosing zone, IV—die.

**Figure 14:**
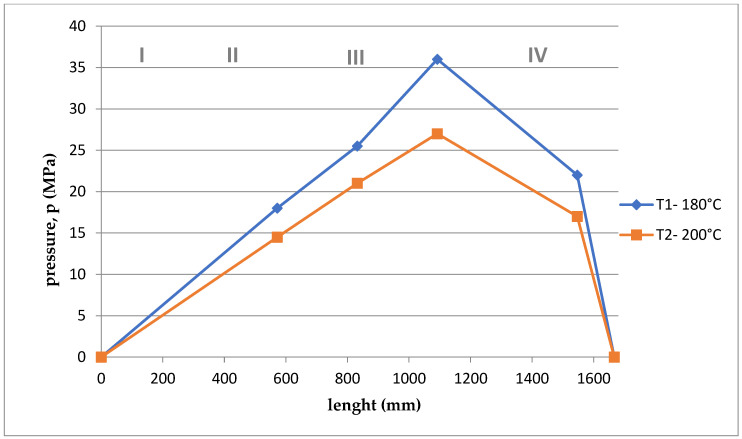
The course of pressure changes during extrusion for two temperatures at 60 rpm. Where: I—supply zone, II—compression zone, III—dosing zone, IV—die.

**Figure 15:**
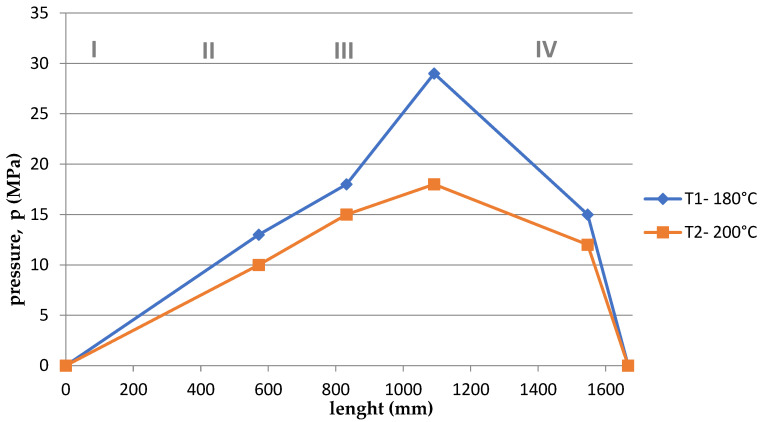
The course of pressure changes during extrusion for two temperatures at 40 rpm. Where: I—supply zone, II—compression zone, III—dosing zone, IV—die.

**Figure 16:**
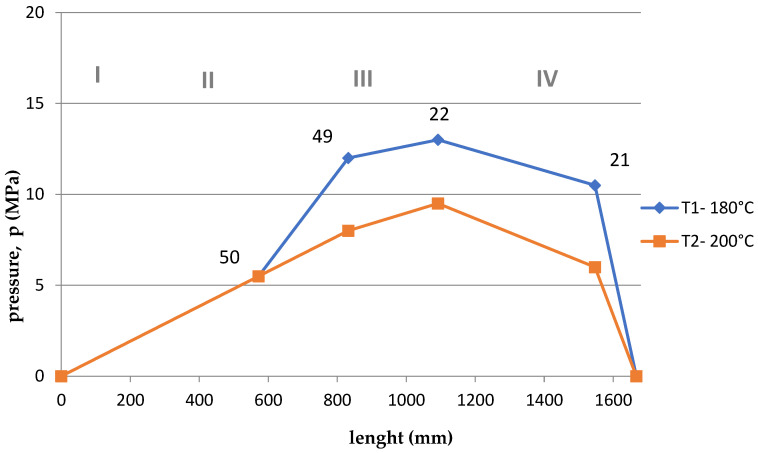
The course of pressure changes during extrusion for two temperatures at 20 rpm. Where: I—supply zone, II—compression zone, III—dosing zone, IV—die.

Based on the arrangement of the sensors on the screw (Figure 1), and considering the division of the screw into 4, appropriate courses of pressure as a function of the length of the screw and the die were developed for the appropriate temperatures T1 = 180 °C and T2 = 200 °C and rotational speeds. The graphs are shown in Figure 11, Figure 12, Figure 13, Figure 14, Figure 15 and Figure 16.

Summing up, it can be stated that:

– With the increase of the rotational speed of the screw, the weight of the PLA sample and the mass yield increased.
– The processing temperature affects the weight of the samples. At a higher temperature, a greater mass of samples was obtained, and thus a greater mass yield.
– The active power increased with the increase in the rotational speed of the screw.
– Processing temperature influences the active power value. At a lower temperature, the active power values were higher. This phenomenon is related, with resistances and clogging of the cochlea.
– As the rotational speed of the screw increased, the current increased.
– The processing temperature affects the current value. At a lower temperature, the current intensity was higher. This phenomenon is also associated with the occurrence of resistances and the so-called clogging of the cochlea.
– As the screw speed increased, the pressure increased.
– The processing temperature affects the pressure value. At a lower temperature, the pressure was higher.
– Viscosity decreased with increasing shear rate and temperature.
– The viscosity of the material increased with increasing pressure.
– Viscosity decreased with increasing volume flow.
– The highest pressure occurred in the third zone of the cochlea, the so-called dosing zone. In addition, the obtained graphs show a rapid pressure drop on the head (IV), which results from its low resistance.
– Observing the graphs in Figure 11, Figure 12, Figure 13, Figure 14, Figure 15 and Figure 16, at lower screw revolutions the graph “expands”, because at lower screw revolutions the residence time of the material on the screw is longer and the material heats up more.

## 4. Conclusions

From the tests of the PLA extrusion process, the results confirming the theoretical assumptions were obtained. They do not differ significantly from the assessment of the extrusion process for the data available in the literature on classic plastics, such as polyethylene (PE) or polypropylene (PP). This suggests that PLA behaves similarly to these plastics in the extrusion process, which allows the use of existing knowledge and experience in processing this biodegradable polymer. The obtained results suggest that PLA is suitable for processing on standard machines. This is important from an industry point of view; it also means that it can potentially be used as a replacement for other plastics in various applications, especially in the packaging industry, as it is biodegradable under industrial composting conditions. Using the obtained data on the rotational speed of the screw, temperature, and other parameters of the PLA extrusion process, computer models can be developed using simulation techniques. They can help predict how changes in process parameters will affect the behavior of the plastic during extrusion, and can help determine the optimal parameters to minimize defects in products, increase productivity, and achieve the desired product quality. Ultimately, they can be used to design the geometry of screws dedicated to PLA, which would allow for more precise adjustment of machines for PLA processing and achieve better efficiency and quality of products.

In conclusion, the results of the study indicate a promising processability of PLA in the extrusion process and the agreement of the obtained results with the knowledge available in the literature, which provides a solid basis for further research and applications of this material.

## Figures and Tables

**Table 1:** Basic properties of Ingeo Biopolymer Ingeo 4043D [45].

Parameter	Value
Density (g/cm^3^)	1.24
Tensile strength (MPa)	127.54
Young’s modulus (MPa)	3585
Softening point (°C)	145–160
Flow rate index (g/10 min)	6
Melting temperature (°C)	210
Drying temperature (°C)	80
Drying time (h)	6
Permissible moisture content (%)	0.025
Supply zone temperature (°C)	180
Temperature in the compression zone (°C)	190
Temperature in the dispensing zone (°C)	200
Die temperature (°C)	190–200

**Table 2:** Pressure values for successive rotations of the screw when measuring T1 = 180 °C.

Screw Rotation n, (rpm)	Sensor Number	Pressure P, (MPa)
20	50	5.5
	49	12.0
	22	13.0
	21	10.5
40	50	13.0
	49	18.0
	22	29.0
	21	15.0
60	50	18.0
	49	25.5
	22	36.0
	21	22.0
80	50	21.5
	49	31.0
	22	36.0
	21	21.0

**Table 3:** List of measurement parameters for processing temperature T1 = 180 °C.

Screw Rotation n, (rpm)	Active Power, (kW)	Amperage, (A)	Average Sample Weight, (g)	Mass Efficiency W, (kg/h)	Pressure P, (MPa)
20	4.0	11.0	38.98	9.36	10.25
40	5.5	17.0	71.21	17.09	18.75
60	7.3	22.0	107.62	25.83	25.38
80	9.0	28.0	143.66	34.48	27.38

**Table 4:** Pressure values for successive rotational speeds of the screw when measuring T2 = 200 °C.

Screw Rotation n, (rpm)	Sensor Number	Pressure P, (MPa)
20	50	5.5
	49	8.0
	22	9.5
	21	6.0
40	50	10.0
	49	15.0
	22	18.0
	21	12.0
60	50	14.5
	49	21.0
	22	27.0
	21	17.0
80	50	18.5
	49	25.0
	22	31.0
	21	19.0

**Table 5:** List of measurement parameters for processing temperature T2 = 200 °C.

Screw Rotation n, (rpm)	Active Power, (kW)	Amperage, (A)	Average Sample Weight, (g)	Mass Efficiency W, (kg/h)	Pressure P, (MPa)
20	3.8	9.0	41.02	9.85	7.25
40	5.0	15.0	77.25	18.54	13.75
60	6.8	19.0	114.90	27.58	19.88
80	8.8	24.0	151.53	36.37	23.38

**Table 6:** Summary of all results for the first temperature T1 = 180 °C.

Screw Rotation n, (rpm)	Average Mass of Samples, (g/s)	Mass Capacity W, (kg/h)	Pressure Drop in the Flow Channel Δp, (MPa)	Volume Flow Q, (cm^3^/s)	Shear Rate -𝛾̇, (1/s)	Viscosity η, (Pa × s)
20	2.60	9.36	10.5	2.10	157.19	1319.98
40	4.75	17.09	15.0	3.83	287.14	722.61
60	7.17	25.83	22.0	5.79	433.94	478.15
80	9.58	34.48	21.0	7.72	579.27	358.19

**Table 7:** Summary of all results for the first temperature T2 = 200 °C.

Screw Rotation n, (rpm)	Average Mass of Samples, (g/s)	Mass Capacity W, (kg/h)	Pressure Drop in the Flow Channel Δp, (MPa)	Volume Flow Q, (cm^3^/s)	Shear Rate -𝛾̇, (1/s)	Viscosity η, (Pa × s)
20	2.73	9.85	6.0	2.21	165.42	1254.34
40	5.15	18.54	12.0	4.15	311.51	666.08
60	7.66	27.58	17.0	6.18	463.29	447.86
80	10.10	36.37	19.0	8.15	610.99	339.59

## Data Availability

Not applicable.

## References

[1] Stasiek J. (2002). Modern technologies and equipment for blowing extrusion of the films. Part I. Blowing extrusion of the films from plastics. Polimery.

[2] Garbacz T., Tor A. (2007). Effect of Porophor Content on the Useful Properties of External Coatings of Cables Obtained by Foaming Extrusion. Polimery.

[3] Haque A.N.M.A., Naebe M. (2023). Material Extrusion of Wool Waste/Polycaprolactone with Improved Tensile Strength and Biodegradation. Polymers.

[4] Dziadowiec D., Matykiewicz D., Szostak M., Andrzejewski J. (2023). Overview of the Cast Polyolefin Film Extrusion Technology for Multi-Layer Packaging Applications. Materials.

[5] Ncube L.K., Ude A.U., Ogunmuyiwa E.N., Zulkifli R., Beas I.N. (2021). An Overview of Plastic Waste Generation and Management in Food Packaging Industries. Recycling.

[6] Stepaniak P., Softić S., Woźniak S., Fabijański M., Nowakowski K. (2023). Properties of polyethylene regranulates made from end-of-life products. Przem. Chem..

[7] Lewandowski A., Wilczyński K. (2022). General model of polymer melting in extrusion process. Polimery.

[8] Rauwendaal C. (2001). Polymer Extrusion.

[9] Wilczyński K., Nastaj A., Wilczyński K.J. (2013). Melting Model for Starve Fed Single Screw Extrusion of Thermoplastics. Int. Polym. Process.

[10] Bawiskar S., White J.L. (1998). Melting Model for Modular Self Wiping Co-Rotating Twin Screw Extruders. Polym. Eng. Sci..

[11] Tadmor Z. (1966). Fundamentals of Plasticating Extrusion. Theor. Model Melting Polym. Eng. Sci..

[12] Vlachopoulos J. (2011). Polymer Rheology and Extrusion.

[13] Tadmor Z., Klein I. (1970). Engineering Principles of Plasticating Extrusion.

[14] Nastaj A., Wilczyński K. (2018). Process Optimization for Single Screw Extrusion of Polymeric Materials—Simulation Studies. Polimery.

[15] Wilczyńskie K.J., Buziak K. (2019). Simulation of single screw extrusion of wood plastic composites based on the on-line pressure measurements. Polimery.

[16] Dhaval M., Sharma S., Dudhat K., Chavda J. (2022). Twin-Screw Extruder in Pharmaceutical Industry: History, Working Principle, Applications, and Marketed Products: An In-depth Review. J. Pharm. Innov..

[17] Wilczyński K.J., Nastaj A., Lewandowski A., Wilczyński K. (2014). A composite model for starve fed single screw extrusion of thermoplastics. Polym. Eng. Sci..

[18] La Galla A., Fiorio R., Erkoç M., Cardon L., D’hooge D.R. (2020). Theoretical Evaluation of the Melting Efficiency for the Single-Screw Micro-Extrusion Process: The Case of 3D Printing of ABS. Processes.

[19] Fabijanski M. (2022). Mechanical Properties of Polylactide Filled with Micronized Chalcedonite. J. Compos. Sci..

[20] Fabijański M. (2022). Effect of multiple processing on the strength properties of polylactide/polystyrene mixture. Przem. Chem..

[21] Foltynowicz Z., Jakubiak P. (2002). Polylactid acid—Biodegradable polymer obtained from vegetable resources. Polimery.

[22] Berzin F., Beaugrand J., Dobosz S., Budtova T., Vergnes B. (2017). Lignocellulosic fiber breakage in a molten polymer. Part 3. Modeling of the dimensional change of the fibers during compounding by twin screw extrusion. Compos. Part A Appl. Sci. Manuf..

[23] Tábi T., Ageyeva T., Kovács J.G. (2021). Improving the ductility and heat deflection temperature of injection molded Poly (lactic acid) products: Comprehensive review. Polym. Test..

[24] He Y., Wu S., Yuen A.C.Y., Huang F., Boyer C., Wang C.H., Zhang J. (2022). Scalable Manufacturing Process and Multifunctional Performance of Cotton Fibre-Reinforced Poly(Lactic Acid) (PLA) Bio-Composites Coated by Graphene Oxide. Polymers.

[25] Garbarski J., Fabijanski M. (2005). Properties of high impact polystyrene flame retarded by magnesium hydroxide and modified with triblock copolymer styrene/butadiene/styrene. Polimery.

[26] Ismail K.I., Pang R., Ahmed R., Yap T.C. (2023). Tensile Properties of In Situ 3D Printed Glass Fiber-Reinforced PLA. Polymers.

[27] Laureto J., Tomasi J., King J.A., Pearce J.M. (2017). Thermal properties of 3-D printed polylactic acid-metal composites. Prog. Addit. Manuf..

[28] Le Duigou A., Castro M., Bevan R., Martin N. (2016). 3D printing of wood fibre biocomposites: From mechanical to actuation functionality. Mater. Des..

[29] Fabijański M. (2016). Study on mechanical properties of phosphogypsum-filled polylactide. Przem. Chem..

[30] Nastaj A., Wilczyński K. (2020). Optimization for Starve Fed/Flood Fed Single Screw Extrusion of Polymeric Materials. Polymers.

[31] Ju Q., Tang Z.P., Shi H.D., Zhu Y.F., Shen Y.C., Wang T.W. (2022). Thermoplastic starch based blends as a highly renewable filament for fused deposition modeling 3D printing. Int. J. Biol. Macromol..

[32] Haider T.P., Völker C., Kramm J., Landfester K., Wurm F.R. (2019). Plastics of the Future? The Impact of Biodegradable Polymers on the Environment and on Society. Angew. Chem.Int. Ed..

[33] Fabijański M. (2019). Mechanical properties of polylactide wood composites. Przem. Chem..

[34] Silva T.F.D., Menezes F., Montagna L.S., Lemes A.P., Passador F.R. (2019). Effect of lignin as accelerator of the biodegradation process of poly(lactic acid)/lignin composites. Mater. Sci. Eng..

[35] Mendoza-Duarte M.E., Estrada-Morena I.A., Lopez-Martinez E.I., Vega-Rios A. (2023). Effect of the addition of different natural waxes on the mechanical and rheological behavior of PLA- A comparative study. Polymers.

[36] Fabijański M. (2019). Mechanical strength and flammability of polylactide. Przem. Chem..

[37] Lebaal N. (2019). Robust low cost meta-modeling optimization algorithm based on meta-heuristic and knowledge databases approach: Application to polymer extrusion die design. Finite Elem. Anal. Des..

[38] Tryznowski M., Soroczyński A. (2020). Use of biodegradable poly(lactic acid) as a binder for molding sands for foundry industry. Przem. Chem..

[39] Fabijański M. (2021). Effect of injection parameters on the mechanical properties of foamed polylactide. Przem. Chem..

[40] Jiang Q., White J.L., Yang J. (2010). A Global Model for Closely Intermeshing Counter-Rotating Twin Screw Extruders with Flood Feeding. Int. Polym. Process..

[41] Zochowski P., Bajkowski M., Grygoruk R., Magier M., Burian W., Pyka D., Bocian M., Jamroziak K. (2021). Ballistic Impact Resistance of Bulletproof Vest Inserts Containing Printed Titanium Structures. Metals.

[42] Hyvärinen M., Jabeen R., Kärki T. (2020). The Modelling of Extrusion Processes for Polymers—A Review. Polymers.

[43] Zhang S., Wang P., Tan L., Huang H., Jiang G. (2015). Relationship between screw structure and properties of recycled glass fiber reinforced flame retardant nylon 46. RSC Adv..

[44] Stepczyńska M., Rytlewski P. (2018). Enzymatic degradation of flax-fibers reinforced polylactide. Int. Biodeterior. Biodegrad..

[45] Ingeo Biopolymer 4043D Technical Data Sheet (data: 01/08/2023). https://www.natureworksllc.com/~/media/Files/NatureWorks/Technical-Documents/Technical-Data-Sheets/TechnicalDataSheet_4043D_3D-monofilament_pdf.pdf.

